# *Plasmodium ovale curtisi* and *Plasmodium ovale wallikeri* in North-West Ethiopia

**DOI:** 10.1186/1475-2875-12-346

**Published:** 2013-09-28

**Authors:** Abebe Alemu, Hans-Peter Fuehrer, Gebeyaw Getnet, Belay Tessema, Harald Noedl

**Affiliations:** 1 Department of Medical Parasitology, School of Biomedical and Laboratory Sciences, College of Medicine and Health Sciences, University of Gondar, Gondar, Ethiopia; 2 Department of Pathobiology, Institute of Parasitology, University of Veterinary Medicine Vienna, Vienna, Austria; 3 Department of Medical Microbiology, School of Biomedical and Laboratory Sciences, College of Medicine and Health Sciences, University of Gondar, Gondar, Ethiopia; 4 Institute of Specific Prophylaxis and Tropical Medicine, Medical University of Vienna, Vienna, Austria

## Abstract

**Background:**

In Ethiopia *Plasmodium falciparum* and *Plasmodium vivax* are the dominant species accounting for roughly 60 and 40% of malaria cases, respectively. Recently a major shift from *P. falciparum* to *P. vivax* has been observed in various parts of the country but the epidemiology of the other human malaria species, *Plasmodium ovale* spp. and *Plasmodium malariae* remains poorly understood. The aim of this study was to assess *P. ovale curtisi* and wallikeri infection in north-west Ethiopia by using microscopy and nested PCR.

**Methods:**

A health institution-based survey using non-probability sampling techniques was conducted at Maksegnet, Enfranze and Kola Diba health centres and Metema hospital in North Gondar. Three-hundred patients with signs and symptoms consistent with malaria were included in this study and capillary blood was collected for microscopic examination and molecular analysis of *Plasmodium* species. Samples were collected on Whatman 903 filter papers, stored in small plastic bags with desiccant and transported to Vienna (Austria) for molecular analysis. Data from study participants were entered and analysed by SPSS 20 software.

**Results:**

Out of 300 study participants (167 males and 133 females), 184 samples were classified positive for malaria (133 *P. falciparum* and 51 *P. vivax*) by microscopy. By species-specific PCR 233 *Plasmodium* spp (95% CI: 72.6-82) were detected and the majority 155 (66.5%, 95% CI: 60.2-72.3) were *P. falciparum* followed by *P. vivax* 69 (29.6%, 95% CI; 24.1-35.8) and 9 (3.9%, 95% CI: 2-7.2) samples were positive for *P. ovale.* Seven of *P. ovale* parasite*s* were confirmed as *P. ovale wallikeri* and two were confirmed as *P. ovale curtisi*. None of the samples tested positive for *P. malariae.* During microscopic examination there were high (16.3%) false negative reports and all mixed infections and *P. ovale* cases were missed or misclassified.

**Conclusion:**

This study indicates that *P. ovale* malaria is under-reported in Ethiopia and provides the first known evidence of the sympatric distribution of indigenous *P. ovale wallikeri* and *P. ovale curtisi* in Ethiopia. Therefore, further studies assessing the prevalence of the rare species *P. ovale* and *P. malariae* are urgently needed to better understand the species distribution and to adapt malaria control strategies.

## Background

Malaria remains one of the leading causes of illness and death in the world [1,2]. *Plasmodium ovale* (first described by Stevens in 1922) [3] is widely distributed across tropical regions in Africa and Asia and is one out of five *Plasmodium* parasite species that causes human malaria [4,5]. Relatively little attention has been paid to ovale malaria, which is considered to be uncommon, mild in clinical presentation and easily treated with the conventional anti-malarial chloroquine [6].

In a study on *P. ovale* conducted by researchers from the UK Malaria Reference Laboratory, London, polymorphisms in six loci were examined in 55 isolates. Two distinct major haplotypes of each locus were identified and these did not recombine in any of the parasites examined. Accordingly, *P. ovale* dimorphism was proposed to reflect the existence of two fully distinct ovale malaria species, which were unexpectedly shown to be broadly sympatric, at the country level, in both Africa and Asia. These two proposed species have been named *P. ovale curtisi* and *P. ovale wallikeri*[7]. Recently both ovale species have been identified in a single individual further strengthening the concept of two distinct *P. ovale* species [8,9].

Malaria remains the leading communicable disease in Ethiopia, accounting for approximately 30% of the overall disability-adjusted life years lost. It is estimated that about 75% of the total area of the country and 68% of the population is at risk of infection [10-12]. In the country *Plasmodium falciparum* and *Plasmodium vivax* are the main species accounting for roughly 60 and 40% of malaria cases [13] and recently there has been a major shift from *P. falciparum* to *P. vivax* in different parts of the country. Despite First report on *P. ovale* in Ethiopia by Armstrong JC IN 1969 [14], the knowledge of the distribution and epidemiology of the other human malaria species, particularly *P. ovale* spp. and *Plasmodium malariae* has never been elucidated and is rather limited in the entire country [15-17].

The global burden of ovale malaria is considered to be light, potentially also because *P. ovale* tends to be mis-and under-diagnosed when using microscopic examination. Difficulties in the diagnosis are partly the result of the low parasite density that is characteristic of malaria caused by ovale malaria parasites [6,18,19]. Species determination at low parasite density is particularly difficult and largely depends on individual skill [20,21]. In developing countries, including Ethiopia, where malaria is diagnosed microscopically by staining thick and thin blood films with Giesma (which despite its inherent limitations remains the gold standard) and where few other diagnostic options are available, *P. ovale* malaria tends to be under-reported.

Therefore, new laboratory diagnostic techniques (such as polymerase chain reaction (PCR)) that can provide higher sensitivity and specificity, without subjective variation, are urgently needed to improve the quality of diagnostics and the understanding of malaria epidemiology [22-24]. The main focus of this study was to assess *P. ovale* species and the ability of microscopists to detect and identify *Plasmodium* species in north-west Ethiopia.

## Methods

### Study area

The study was conducted at three health centres (Maksegnet, Enfranze and Kola Diba) and one hospital (Metema hospital) in North Gondar, northwest Ethiopia. These areas are endemic for malaria and the altitude of the districts ranges from 1,750 to 2100 m above sea level. Malaria is the most prevalent seasonal disease in the area, ranking second among all reported diseases seen at the health centres. October to December is the peak transmission season for *P. falciparum*. Both *P. vivax* and *P. falciparum* are known to co-exist in the area whereas other *Plasmodium* species have not been reported in the past two decades from any of the four sites. The only reports of *P. ovale* in Ethiopia date back to the 1960s [14]*.*

### Study design and inclusion criteria

A health institution-based survey using non-probability sampling techniques was conducted at three health centres and one hospital in North Gondar, north-west Ethiopia. Patients with signs and symptoms consistent with malaria (for which diagnostic blood films are requested at any of the participating health institutions) were included in this study. Males and females, of any age, with signs and symptoms consistent with malaria and willingness to participate and sign the informed consent, were used as inclusion criteria.

### Sample size and sampling techniques

Convenience sampling technique was used to recruit study participants for this study. A total of 300 suspected malaria patients were included from March 2 to April 26, 2013. Participants were 184 microscopy-confirmed cases and 116 febrile patients suspected of potentially having malaria and who were negative by microscopy. A structured questionnaire was used to obtain sociodemographic information (sex, age, residence, ethnicity, religion, and clinical data).

### Blood sample collection and microscopic malaria parasite examination

From each participant a small amount of capillary blood was collected by finger prick after obtaining informed consent. The total amount of blood was less than 1 mL. Peripheral smear examination of well-prepared and well-stained blood films remains the gold standard for confirming the presence of malaria parasites. Thick and thin smears prepared from the peripheral blood were used for this purpose and for each patient two slides were prepared.

Before staining the thin blood films were fixed in methanol for 30 sec. Then smears used for study purposes were stained with 3% Giemsa solution for 30 min, whereas the staining of the routine smear followed the standard techniques established at the respective health facility. Examination of the blood films was conducted according to standardized World Health Organization (WHO) protocols. Thick films were used for the detection of the presence of parasites and parasite species identification was done by using thin films under 100 × oil immersion objective.

### Sample collection for molecular analysis

Two blood spots were collected from each participant, transferred onto filter paper (Whatman 903) and labelled with the participant’s study code and date. Each filter paper was dried individually, carefully avoiding any chance of contamination. The samples were then stored in small plastic bags with desiccant and transported to the Institute of Specific Prophylaxis and Tropical Medicine, Medical University of Vienna (MUV), Vienna, Austria for molecular analysis.

### DNA isolation and purification

A modified chelex-based DNA extraction method using the InstaGene Whole Blood Kit (Bio-Rad Laboratories, Hercules, CA, USA) was used for the extraction and purification of *Plasmodium* DNA from the blood spots on filter paper. To ensure comparable quantities of blood, samples of exactly 4 mm diameter were punched out of the blood spots. Blood spots were soaked overnight in a tube at 4°C with 100 μl phosphate-buffered saline (PBS). The tube was centrifuged at 13,000 revolutions per minute (rpm) for 2 min. After discarding the supernatant, 100 μL of PBS was added to the tube and the sample was centrifuged at 13,000 rpm for 2 min to wash the sediment. After discarding the supernatant, 60 μL of InstaGene Matrix was added. The tube was incubated for 8 min at 70°C, vortexed for 15 sec, boiled for 4 min, and vortexed for 15 sec. Finally, the tube was centrifuged at 13,000 rpm for 1 min and the supernatant was aspirated and purified again with InstaGene Matrix as described. The supernatant was then used for the PCR. If the PCR result was negative, the supernatant was purified again for up to three times.

### Parasite detection by nested PCR

Nested PCR assay was performed for all samples as reported previously [22,24-26]. Each 50 μl reaction mixture for nest 1 amplifications contained 5 μl of DNA template, 5 μl (10 μM) of each primer (rPLU 1 and rPLU 5), 2 mM MgCl_2_, PCR buffer, 125 μM of each dNTP, and 1 unit of GoTaq DNA polymerase (Promega, Madison, WI, USA). Nest 1 amplification conditions were as follows: initial denaturation 94°C for 4 min; 25 cycles (1 min denaturation at 94°C, annealing at 58°C for 2 min, extension at 72°C for 2 min); and a final extension at 72°C for 4 min; 2.5 μl of the nest 1 amplification product served as the DNA template for each of the 25 μl nest 2 amplifications.

The concentration of the nest 2 primers and other constituents were identical to nest 1 amplifications. Nest 2 amplification conditions were identical for all species-specific nest 2 PCRs to those of nest 1 except for the genus-specific primers (rPLU 3 and 4; annealing: 62°C). In all experiments, a negative control of water and known positive controls were run with the samples. In the nested PCR, the genus-specific primers rPLU1 and rPLU5 for the first PCR and rPLU3 and rPLU4 were used for the second PCR. Whenever this genus-specific nested PCR gave positive results, species-specific nest 2 PCRs were performed for species determination using the following internal primer pairs: rFAL1 and rFAL2 for *P. falciparum*, rVIV1 and rVIV2 for *P. vivax*, rMAL1 and rMAL2 for *P. malariae* , rOVA1WC and rOVA2WC for both *P. ovale* species [22,24-26].

The oligonucleotides were obtained from Microsynth (Microsynth AG, Balgach, Switzerland). All samples giving positve signals for *P. ovale* spp were further analysed to species level using the primers rOVA1/rOVA2 specific for *Plasmodium ovale curtisi* and rOVA1v/rOVA2v for *Plasmodium ovale wallikeri*[25,26]. The PCR products of nest 2 amplifications were analysed by gel electrophoresis with 2% agarose and Midori Green staining. The individual interpreting the PCR results was blinded to the results of microscopy.

### Sequence analysis

The amplicons of *P. ovale*-positive samples were further analysed by sequencing for species confirmation. PCR products were purified with ExoSap-IT® (GE Healthcare, Buckinghamshire, UK) and sequenced using the BigDye Sequencing Kit and an automatic 310 ABI PRISM sequencer (PE Applied Biosystems, Weiterstadt, Germany).

### Data analysis

Data were checked for their completeness before entering for analysis; then all the data from study participants were imported into Excel and analysed by SPSS 20 program software. Descriptive statistics was used to assess the distribution of the sociodemographic characteristics and independent variables as well as frequencies and proportion of malaria-positive samples.

### Ethical consideration

This study was expected to pose only minimal risks to the participants. The study protocol was reviewed and approved by the Institutional Ethical Review Board of the University of Gondar. Written informed consent in local language was obtained from each participant and/or his/her legal representative. For patients who cannot read or write the consent form was read in the local language and a thumbprint was collected instead of a signature. Patients who were positive for malaria parasites received standard treatment according to national treatment guidelines.

## Results

### Sociodemography characteristics of the study subjects

Out of a total of 300 study participants, 167 (55.7%) were male and 133 (44.3%) were female. The majority of the respondents (91.7%) were of Amhara ethnicity and orthodox christianity was the predominant religion in the area accounting for 93.7%. In total 37.3% of the study participants were illiterate and the majority (61%) were in the age group 15 to 44 years (Table 1).

**Table 1:** Sociodemography characteristics of malaria-suspected febrile patients in three health centres and one hospital in North Gondar, northwest Ethiopia, 2013 (n = 300)

**Variables**	**Frequency**	**Percentage**
**Sites**		
Kola Diba	65	21.7
Maksegnet	72	24
Enfranze	113	37.7
Metema	50	16.6
**Gender**		
Male	167	55.7
Female	133	44.3
**Age**		
<5	30	10
5-14	61	20.3
15-44	183	61
>44	26	8.7
**Religion**		
Orthodox	281	93. 7
Muslim	13	4.3
Others	6	2
**Ethnicity**		
Amhara	275	91.7
Tigrea	21	7
Others	4	1.3
**Educational status**		
Illiterate	112	37.3
Read and write	100	33.3
Elementary completed and above	88	29.4

### Comparison of malaria parasite detection by microscopy and nested PCR

A total number of 184 (133 *P. falciparum* and 51 *P. vivax*) samples classified as positive by microscopic examination were confirmed as being positive by PCR. In addition, 49 samples that were classified as negative by microscopy were positive in the genus-specific nested PCR. Only two (0.7%, 95% CI: 0.2-2.4) samples that were read as positive by microscopy were considered false positive. The result indicates that 16.3% (49/300) of the malaria cases were missed by microscopy. By species-specific PCR, 233 *Plasmodium* spp (95% CI: 72.6-82) were detected and the majority 155 (66.5%, 95% CI: 60.2-72.3) were *P. falciparum* followed by *P. vivax* 69 (29.6%, 95% CI; 24.1-35.8) and nine (3.9%,) samples were positive for *P. ovale.* Furthermore, nine (3.9%, 95% CI: 2-7.2) samples gave positive results for *P. ovale* spp, but all examined samples were negative for *P. malariae* (Table 2)*.*

**Table 2:** Comparison of malaria parasite detection by microscopy and nested PCR in three health centres and one hospital in North Gondar, northwest Ethiopia, 2013 (n = 300)

**Sites**	**Total slide examined**	**Microscopically confirmed malaria parasites**							
		**Pf**	**Pv**	**Po**	**Pm**	**Pf + Pv**	**Pf + Po**	**Pv + Po**	**Pf + Pv + Po**
Kola Diba	65	27	9	-	-	-	-	-	-
Maksegnet	72	25	11	-	-	-	-	-	-
Enfranze	113	60	28	-	-	-	-	-	-
Metema	50	21	3	-	-	-	-	-	-
Total	300	133	51						
**Sites**	**Total sample analysed**	**Result of species specific nested PCR**							
		**Pf**	**Pv**	**Po**	**Pm**	**Pf + Pv**	**Pf + Po**	**Pv + Po**	**Pf + Pv + Po**
Kola Diba	65	21	8	3	-	6	2	-	-
Maksegnet	72	30	16	2	-	2	-	-	-
Enfranze	113	63	28	1	-	3	-	-	-
Metema	50	26	5	-	-	1	1	-	-
Total	300	140	57			12	3		

Note: Pf = *P. falciparum* ; Pv = *P. vivax* ; Pm = *P. malariae* ; Po = *P. ovale.*

All positive samples (184) were mono-infections and all mixed *Plasmodium* infections were missed and all *P. ovale* were missed or wrongly classified by microscopy. By the PCR results, out of 233 positive samples 15 (6.4%, 95% CI: 3.9-10.4) samples were mixed malaria infections, which indicates that the majority of 218 (93.6%, 95% CI: 89.7-96.1) cases were mono-infections.

(Table 2) Additionally, PCR analysis revealed 49 (16.3%, 95% CI: 12.6-20.9) cases gave false negative results by microscopy and only two (0.7%, 95% CI: 0.2-2.4) false positive reporting of microscopic slides from febrile patients. In addition to false negative and positive report, there was wrong classification of *Plasmodium* species by microscopy. Three *P. ovale* parasites were reported as *P. vivax*, two were reported as *P. falciparum*, eight *P. falciparum* as *P. vivax* and four *P. vivax* as *P. falciparum.*

### Distribution of *Plasmodium ovale* species among symptomatic patients in North Gondar

Nine out of 300 (3%) blood samples collected from symptomatic patients presenting febrile illnesses were positive for *P. ovale* spp, out of which the majority, seven cases (77.7%) were confirmed as *P. ovale wallikeri* and only two cases (23.3%) were confirmed as *P. ovale curtisi*. Four samples positive for *P. ovale* in PCR were confirmed by DNA sequencing (GenBank Accession numbers: KF536873 and KF536874–*P. ovale curtisi*; KF536875 and KF536876–*P. ovale wallikeri*). However, none were diagnosed as *P. ovale*, as *P. ovale* was not known to be endemic in Ethiopia, including in the study areas and, therefore, it was not adequately considered by the microscopists (Table 3). *Plasmodium ovale* is distributed in all sites in north Gondar with high frequency in Kola Diba.

**Table 3:** **Distribution of *Plasmodium ovale *species among symptomatic patients in North Gondar, northwest Ethiopia, 2013 (n = 300)**

**Sites**	**Total samples analysed**	***Plasmodium ovale *species**	
		***P. ovale wallikeri***	***P. ovale curtisi***
Kola Diba	65	3	2
Maksegnet	72	2	
Enfranze	113	1	
Metema	50	1	

## Discussion

Malaria remains a leading cause of infectious disease in Ethiopia, accounting for approximately 30% of the overall disability-adjusted life years lost. It is estimated that about 75% of the total area of the country and 68% of the population is at risk of infection. In the country, the two *Plasmodium* species, *P. falciparum* and *P. vivax*, are the main and dominant species accounting for roughly 60 and 40% of malaria cases, respectively [13]. Recently, there has been a major shift from *P. falciparum* to *P. vivax* in different parts of the country. However, the epidemiology of the *P. ovale* spp remains poorly understood and there are no recent data on the distribution of this pathogen in Ethiopia (including the study area) [15-17].

The PCR results of this study indicate that *P. falciparum* (66.5%) remains the predominant species followed by *P. vivax* (29.6%) and *P. ovale* (3.9%) at the study sites. The majority (93.6%) of infections were mono-infections. This result is concordant with previous studies elsewhere in Ethiopia and the report of the Ethiopian Federal Ministry of Health, in which *P. falciparum* is the predominant malaria circulating in the country followed by *P. vivax.* On the other hand, this finding contradicts previous results, which indicated the shifting of *P. vivax* in the study area and different parts of Ethiopia and no report of *P. ovale* cases recently [15-17]. However, virtually all previous studies conducted in Ethiopia are based on microscopic data only, whereas this study used a highly sensitive nested PCR assay with a documented limit of detection of only six parasites/μL [27].

The 3.9% positive rate for *P. ovale* infections among suspected malaria cases is the first report of *P. ovale* from this part of the country. Until relatively recently the distribution pattern of *P. ovale* was considered to be limited to tropical regions in sub-Saharan Africa, Indonesia (e.g., Timor, Flores, West Papua) and the Philippines [6], but not from health facilities treating malaria in Ethiopia, possibly due to its tertian periodicity, typically low parasitaemia and morphological resemblance (when fixed and stained) to *P. vivax*[28]. In analogy to previous studies, three *P. ovale* cases were misclassified as *P. vivax* and two were reported as *P. falciparum,* suggesting difficulty in the diagnosis of *P. ovale* by microscopic examination in the study area where this pathogen was previously not known to be present and most microscopists are neither trained to detect nor aware of the presence of other species [12].

*P ovale-*positive samples were further analysed with specific PCRs and/or sequenced to classify *P. ovale* species in the study area and the majority (77.7%) were confirmed as *P. ovale wallikeri* as compared to 23.3% that were identified as *P. ovale curtisi*. This finding is in line with the previous report by Sutherland *et al*. [7], that showed the perfect linkage between the dimorphic forms at each locus and led to the introduction and segregation of *P. ovale* into two species, namely *P. ovale curtisi* (former classic type) and *P. ovale wallikeri* (former variant type). This finding is the first of its kind in Ethiopia and is in line with previous reports from South Asia in which both *P. ovale curtisi* and *P. ovale wallikeri* occur sympatrically in the Chittagong Hill Tracts, Bangladesh [29,30]. These two *P. ovale* species are morphologically identical and cannot be differentiated by microscopy [31]. Even molecular analysis has previously resulted in inconsistent results [32].

The nested PCR analysis suggests a relatively high false negative rate of 16.3% with only 0.7% false positive cases at microscopy as well as misclassification of *Plasmodium* species. (e.g., *P. ovale* as *P. vivax* or *P. falciparum*; *P. falciparum* as *P. vivax*; *P. vivax* as *P. falciparum*). Although microscopy remains the gold standard for malaria diagnosis in the field, the limits of detection may significantly differ between microscopists and have previously been estimated to range from 50 to 100 parasites/μl under field conditions [23]. Microscopy depends on well maintained equipment, uninterrupted supply of good-quality reagents, trained staff, and good-quality monitoring and supervisory systems. Maintaining a quality-assured microscopy service is a major challenge, even for health centres and district hospitals.

The consequences of misdiagnosis of malaria are felt not only at health centres, but also at national levels. Individuals wrongly diagnosed with malaria are exposed to unnecessary side-effects of drugs, and the true causes are not recognized or treated. This scenario is likely to lead to prolonged and worsening illness with loss of income or productivity, and repeated visits to health providers [21]. This study tried to assess the existence and genetic diversity of *P. ovale* parasites while at the same time assessing the ability of microscopists to detect and identify *Plasmodium* species. In spite of its limitations (mainly the inability to determine true prevalence), this study assessing of *P. ovale spp.* is the first of its kind in Ethiopia and provides important information regarding the *Plasmodium* species spectrum and distribution in the country as the basis for larger scale surveys.

## Conclusion

Despite of the high prevalence of malaria in Ethiopia and in the study areas, there had previously been no report of the presence of either *P. ovale wallikeri* or *P. ovale curtisi*. On the one hand this study provides *P. ovale* malaria is under-reported and the first known evidence of the sympatric distribution of indigenous *P. ovale wallikeri* and *P. ovale curtisi* in northwest Ethiopia and is meant to provide the Ethiopian government and researchers with guidance regarding the presence of *Plasmodium* specie*s* other than *P. falciparum* and *P. vivax*. At the same time it highlights the importance of adequate training for health staff involved in malaria diagnosis and the inherent limitations of microscopic diagnosis.

## Competing interests

The authors declare that they have no competing interests.

## Authors’ contributions

AA and HN conceived the study. AA and HFP undertook statistical analysis, undertook molecular analysis of the samples and drafted the manuscript. GG and BT contributed in proposal writing, blood sample collection and microscopic diagnosis of malaria slides. All authors contributed to the writing of the manuscript and approved the submitted version of the manuscript.
